# Psoriatic arthritis in psoriasis: optimizing the current screening system for psoriatic arthritis based on serum data from U.S. and Chinese populations

**DOI:** 10.3389/fimmu.2024.1497713

**Published:** 2024-12-10

**Authors:** Zheng Lin, Si-yi Pan, Yue-yi Shi, Xuan Wu, Yuan Dou, Ping Lin, Yi Cao

**Affiliations:** ^1^ Department of Dermatology, First Clinical Medical College of Zhejiang Chinese Medical University, Hangzhou, Zhejiang, China; ^2^ Department of Nephrology, Hangzhou Traditional Chinese Medical (TCM) Hospital Affiliated to Zhejiang Chinese Medical University, Hangzhou, Zhejiang, China; ^3^ Department of Geriatrics, The Third Hospital of Hangzhou, Hangzhou, Zhejiang, China

**Keywords:** psoriasis, psoriatic arthritis, screening, PSAII, machine learning

## Abstract

**Background:**

Psoriatic arthritis (PSA) is an inflammatory joint disease associated with psoriasis (PSO) that can be easily missed. Existing PSA screening tools ignore objective serologic indicators. The aim of this study was to develop a disease screening model and the Psoriatic Arthritis Inflammation Index (PSAII) based on serologic data to enhance the efficiency of PSA screening.

**Method:**

A total of 719 PSO and PSA patients from the National Health and Nutrition Examination Survey (NHANES) (as training set and test set) and 135 PSO and PSA patients who were seen at The First Affiliated Hospital of Zhejiang Chinese Medical University (as external validation set) were selected, 31 indicators for these patients were collected as potential input features for the model. Least Absolute Shrinkage and Selection Operator (LASSO) was used to identify PSA-related features. Five models of logistic regression (LR), random forest, k-nearest neighbor, gradient augmentation and neural network were developed in the training set using quintuple cross validation. And we developed PSAII based on the results of LASSO regression and weights of logistic model parameters. All performance metrics are derived on the test set and the external validation set.

**Results:**

Five variables were selected to build models, including age, lymphocyte percentage, neutrophil count, eosinophilic count, and C-reactive protein. In all established models, the LR model performed the best, with an Area Under Curve (AUC) of 0.87 (95% confidence interval (CI): 0.83-0.90) on the test set; on the external validation set the AUC was 0.82 (95%CI: 0.74-0.90). The PSAII formula was PSAII = percentage of lymphocytes × C-reactive protein/(neutrophil count × eosinophilic count × 10). The AUC of PSAII in the test is 0.93 (95%CI: 0.88-0.97), and the cutoff value is 18. The AUC of the external validation set is 0.81 (95%CI: 0.72-0.89).

**Conclusions:**

This study developed and validated five models to assist screening for PSA by analyzing serum data from NHANES and Chinese populations. The LR model demonstrated the best performance. We created PSAII for PSA screening. However, the high false positive rate of PSAII makes it necessary to combine it with other PSA screening tools when applied.

## 1 Introduction

Psoriasis (PSO) is a chronic, immune-mediated skin disease characterized by the appearance of erythematous, scaly, bleeding, and itchy patches of skin that affects approximately 2% of the U.S. population (1), affecting approximately 0.11% of the population in East Asia (2). The pathogenesis of PSO is relatively complex and is influenced by the interplay of genetic loci, immune imbalances, environmental triggers, and other factors (3). As PSO has been studied in depth, it has been recognized as a systemic disease. Compared with the general population, patients with PSO have a significantly increased risk of developing comorbidities such as psoriatic arthritis (PSA), metabolic syndrome, and cardiovascular disease (4). PSA is an inflammatory joint disease associated with PSO, characterized by chronic progressive musculoskeletal inflammation that affects approximately 20% of PSO patients (5). The burden of PSA on patients is significant, including, but not limited to, irreversible joint damage, high treatment costs, and stigmatizing impacts (6–8), which makes the prevention and treatment of PSA a topic of global medical concern.

According to a cross-sectional study, in more than 80% of PSA patients, skin symptoms precede joint symptoms by more than ten years (9). Therefore, screening for PSA in patients with PSO is reasonable and necessary. However, due to the insidious and slow onset of PSA, and also due to the lack of effective and easy screening tools for PSA (10), screening for PSA is difficult. According to one epidemiologic study, approximately 41% of PSA cases are missed by physicians (11), and approximately 50% of patients with PSA have disease symptoms well beyond the minimum disease activity of PSA when they first receive treatment (12). Delayed diagnosis leads to poor prognosis of PSA patients, and early intervention can benefit patients with PSA from disease manifestations and prognosis (13), which makes the development of easy screening tools for PSA a priority in the prevention and treatment of PSA.

The events leading to progression to PSA are unknown, with the central role of genetic susceptibility and activation of inflammatory pathways being the influences that have received the most attention (14). In addition, DNA methylation, microbial ecological dysregulation, and biomechanical stress have been increasingly reported to play a role in PSA pathogenesis in recent years (15–17). Polyarticular pain, elevated C-reactive protein, elevated lymphocyte levels, nail involvement and severe PSO have been suggested as possible risk factors of PSA. Cardiovascular disease and uveitis are comorbidities associated with PSA, which share common pathophysiologic pathways (18–20). Despite these findings, there is still a lack of validated soluble biomarkers for diagnosing or predicting the development of PsA in clinical practice (21). This has led current PSA screening tools, such as the Psoriatic Arthritis Screening and Evaluation Tool (PASE), the Psoriasis Epidemiology Screening Tool Questionnaire (PEST), and the Toronto Psoriatic Arthritis Screen (ToPAS), to limit the focus of screening to only the symptoms and signs, which may be one of the reasons for the lack of efficacy of these instruments (22). In addition, due to the insidious onset of PSA, subjective self-reporting by patients has become an important component of PSA screening, which may elevate the rate of underdiagnosis of PSA. In PSA screening, screening by more objective indicators by those with medical knowledge will undoubtedly enhance screening efficiency. Therefore, more multidimensional and objective PSA screening tools need to be developed.

With the understanding and development of inflammation, more and more composite inflammatory indices have been recognized as more comprehensively reflecting the nature of inflammation, such as the systemic immunoinflammatory index (SII), neutrophil/lymphocyte ratio (NLR), platelet/lymphocyte ratio (PLR) (23). Among them, SII and systemic inflammatory response index (SIM) have been shown to be associated with PSO in several studies (24, 25). However, in the case of PSA, as far as we know, there have been very few studies in this area. Therefore, one of the goals of our study is to test the potential of these composite inflammatory indices to screen patients with PSA. Based on this, we will utilize machine learning to build a PSA screening model based on serum data and attempt to develop a psoriatic arthritis inflammation index (PSAII) with better screening performance, aiming to improve PSA screening efficacy among PSO patients.

## 2 Methods

### 2.1 Data collection from the National Health and Nutrition Examination Survey

#### 2.1.1 Study population

This study was conducted according to the NHANES database. All NHANES protocols were approved by the NCHS Research Ethics Review Board (Protocol #98- 12, Continuation of Protocol #2005-06, Continuation of Protocol #2011-23, http://www.cdc.gov/nchs/nhanes/irba98.htm) and informed consent was obtained at participant enrollment. A total of 88402 participants from eight NHANES cycles (2003-2004, 2005-2006, 2009-2010, 2011-2012, 2013-2014, 2015-2016, 2017-2020, 2021-2023) participated in this study. Inclusion criteria included: (1) age >18 years and (2) Participants who self-reported having PSO or PSA. Exclusion criteria included: (1) Participants with more than 1/3 missing data; (2) self-reported having other diseases that may affect metabolism (e.g., hypothyroidism, tumors, etc.); (3) self-reported having other diseases that may affect inflammatory markers (e.g., pneumonia, gastroenteritis, etc.); (4) self-reported having other diseases that may cause joint pain (e.g., rheumatoid arthritis, gout, etc.). Ultimately, a total of 574 patients with PSO and 145 with PSA were included in this study.

#### 2.1.2 Evaluation of PSO and PSA

PSO was defined if the participant answered yes to the questions “Have medical personnel ever told you that you have PSO” or “Has a doctor or other health care professional ever told you that you have PSO”. PSA was defined as if the participant answered yes to the question “Do you have arthritis” and “PSA” to the question “What type of arthritis”. Participants who chose to refuse to answer or answered “did not know” were excluded from the study.

#### 2.1.3 Covariate

We potential influencing factors that may affect PSO and PSA were evaluated based on the available literature. These variables included age, sex (male and female), race/ethnicity (Mexican American, other Hispanic, non- Hispanic white, non-Hispanic black, other race/multiracial), education, body mass index (BMI), smoking history, alcohol use, diabetes, hypertension, and serological markers. Participants who responded affirmatively to the question “smoked at least 100 cigarettes in my lifetime” were considered to have a history of smoking. Participants who answered yes to the question “More than 12 drinks per year” were considered to have a history of alcohol use. Participants who answered yes to the question “Doctor told you have diabetes” were considered to have diabetes. Participants who answered yes to the question “Ever told you had high blood pressure” were considered to have hypertension. According to the question “Is Psoriasis little or extensive?”, graded patients with psoriasis into four levels of severity, including “Little or no psoriasis,” “Only a few patches (that could be covered by one or two palms of (your/his/her) hand)”, Scattered patches (that could be covered between three and ten palms of hand), “Extensive psoriasis (covering large areas of the body, that would be more than ten palms of hand)”.

Peripheral blood samples from NHANES participants were analyzed at the Mobile Examination Center (MEC) using a Beckman Coulter HMX Hematology Analyzer, and complete blood counts (WBCs) were classified using the VCS technique. The DxC800 measured various biochemical markers in the serum or plasma using kinetic rate, enzyme rate, and enzyme conductivity assays. Twenty serological markers were ultimately enrolled in the study, including the white blood, leukocyte count, neutrophil ratio, lymphocyte ratio, monocyte ratio, eosinophil ratio, basophil ratio, ultrasensitive C protein, albumin, etc.

### 2.2 Data collection from The First Affiliated Hospital of Zhejiang Chinese Medical University

#### 2.2.1 Study population

We conducted a retrospective study including 98 patients with PSO and 37 patients with PSA who attended The First Affiliated Hospital of Zhejiang Chinese Medical University from January 2022 to January 2024. The protocol was approved by the Ethics Review Committee of Zhejiang Provincial Hospital of Traditional Chinese Medicine, and our hospital’s ethics committee waived our informed consent because we only reviewed the existing database. The inclusion criteria for this study were as follows: (1) age >18 years; (2) those who were clinically diagnosed with PSO or PSA. The exclusion criteria of this study were as follows: (1) those with incomplete case data; (2) those who suffered from other diseases that might affect metabolism (e.g., hypothyroidism, tumors, etc.) at the time of admission; (3) those who suffered from other diseases that might affect inflammatory indexes (e.g., pneumonia, gastroenteritis, etc.) at the time of admission; (4) those who suffered from other diseases that might cause joint pain (e.g., rheumatoid arthritis, gout, etc.); (5) those who were treated in January prior to the admission with hormones, immunosuppressants, biologics inhibitors, and patients treated with biologics. Finally, 98 patients with PSO and 37 patients with PSA were included in this study.

#### 2.2.2 Evaluation of PSO and PSA

The diagnosis of PSO was made by experienced dermatologists who were unaware of the current study, with primary reference to the Chinese PSO diagnostic criteria (26), which was made based on the typical lesion presentation and dermoscopy. The typical lesion presentation was characterized by lesions of infiltrative erythema covered with white or silvery-white scales with wax droplets, membranous phenomena, and punctate hemorrhages. Dermoscopy: The typical dermoscopic features of psoriasis vulgaris are punctate and globular blood vessels uniformly distributed on a red background with diffuse white scales. When magnified more than 50 times, punctate and globular blood vessels appear as clusters of capillaries or glomeruloids.

The diagnosis of PSA is performed by an experienced dermatologist and rheumatologist. All PSA patients were diagnosed with PSA for the first time, and the criteria referred to the PSA diagnosis and treatment guideline released by the Chinese Physicians Association in 2022 (27), which recommended the CASPAR classification criteria. On the basis of the presence of inflammatory arthritis, those who fulfilled three of the following five were diagnosed with PSA: (1) Evidence of PSO; (2) Typical PSO nail changes; (3) Negative rheumatoid factor; (4) Current or past history of inflammation of the toes; (5) Imaging evidence of new bone formation in the proximal osteoarthritis.

#### 2.2.3 Covariate

From all the participants, we collected the following clinical and laboratory parameters: age, sex, weight, height, BMI, smoking history, alcohol consumption history, history of diabetes, history of hypertension, body surface area (BSA) of psoriasis, blood leukocyte count, neutrophil ratio, lymphocyte ratio, monocyte ratio, eosinophil ratio, basic phagocyte ratio, absolute neutrophil count, absolute lymphocyte count, ultrasensitive reactive C protein. All serological indices were collected according to the following criteria: venous blood samples were collected from the anterior elbow vein of all subjects after a one-night fast (at least 8 hours) and analyzed in our central laboratory according to standard laboratory procedures. Psoriasis severity was divided into four grades based on BSA score, includes: little or no psoriasis (BSA <=2), only a few patches (2<BSA<=4), scattered patches (4<BSA<=10), extensive psoriasis (BSA>10).

### 2.3 Formula for calculating complex inflammatory indicators

Some composite inflammation indices were selected to evaluate their potential to screen PSA patients, including SII, NLR, PLR, Lymphocyte to monocyte ratio (LMR), Neutrophil to platelet ratio (NPR), SIM, Platelet to albumin ratio (PAR), CRP albumin lymphocyte (CALLY), which was calculated as follows: SII = Platelet count × neutrophil count/lymphocyte count; NLR = Neutrophil count/lymphocyte count; PLR = Platelet count/lymphocyte count; LMR = Lymphocyte count/monocyte count; NPR = Neutrophil count/platelet count; SIM = Monocyte count × neutrophil count/lymphocyte count; PAR = Platelet count/albumin; CALLY = Albumin × Lymphocyte/(C-reactive protein ×10).

### 2.4 Statistical method

#### 2.4.1 Data weighted and univariate analysis

In general, based on the nature of complex multistage sampling in the NHANES database, adjustments should be made using specific sample weights, clustering, and stratification. However, in this study, firstly, PSO and PSA participants came from multiple years, with some year stages having information only on PSO participants and some year stages having information only on PSA patients, so the data included in this maneuver are not fully representative of the U.S. population. Second, the main purpose of this study was to classify PSA and PSO based on the serum data of PSA and PSO participants. In summary, in this study, we believe that the use of sample weights is not necessary.

We performed univariate analysis and correlation analysis for all variables, normal continuous data were expressed as mean ± standard deviation, skewed continuous data were expressed as median (upper quartile, lower quartile), and categorical parameters were expressed as number of patients (percentage). Continuous data were tested for normality by Shapiro’s test and histograms and were considered normal at p>0.05. Differences between groups were calculated by t-test for continuous normal data, Wilcoxon M-W test for continuous skewed data, and Pearson’s chi-square test for non-parametric data, and differences were considered statistically significant if the p-value was <0.05. All data in this paper were analyzed using R version 4.3.1 (R Foundation for Statistical Computing, Vienna, Austria) to analyze the data.

There were missing values for some variables from the NHANES database, and to solve this problem, we used random forest regression to estimate the missing data. Data from Zhejiang Provincial Hospital of Traditional Chinese Medicine had good completeness and did not require missing values to be filled in.

For ease of calculation, the data processing steps are as follows: Sex was coded as male 1 and female 0; dichotomous variables (smoking history, drinking history, etc.) were coded with 1 for yes and 0 for no. Ethnicity was coded with 1 for Mexican American, 2 for other Hispanic, 3 for non-Hispanic white, 4 for non-Hispanic black, and 5 for other race/multiracial.

#### 2.4.2 Sample size calculation and division of training and test sets

In this paper, sample size is calculated using the pmsampsize package for R, which implements the previously published specification for model sample size calculation in the BMJ (28), with the following specific parameters (type = “b”, cstatistic = 0.90, parameters = 5, prevalence = 0.25). The calculation shows that our training set needs at least 289 examples of PSO data and 73 examples of PSA data.

Therefore, we used the data collected from the NHANES database as the training set and test set using five-fold cross-validation for developing the model and PSAII. In the cross-validation, 719 cases of data were randomly divided into 5 pieces, one of which was selected as the test set (144 cases), and the other 4 pieces as the training set (545 cases). Used the data from The First Affiliated Hospital of Zhejiang Chinese Medical University as the external validation set. The final performance index was taken as the average of all the generated performance indicators, aiming to prevent overfitting of the model and ensure the accuracy of the estimation of key parameters in the prediction model. All data included in the model were normalized before calculation by maximum-minimum normalization with the following formula: E(x) = (x- min)/(max - min).

#### 2.4.3 Development of disease screening models and PSAII

The model endpoint in this study was defined as the onset of PSA, and a total of 31 potential risk factors were collected. These indicators were assessed for correlation and ROC curves for individual factors were plotted before modeling was performed, indicators with high AUC values were considered to have potential for inclusion in the model, and those with high correlation would be excluded from the model. The Least Absolute Shrinkage Selection Operator (LASSO) algorithm was used in this study to select metrics highly correlated with PSA. In this paper, LASSO regression using the glmnet package for R was used to screen potential risk factors and ten-fold cross-validation was performed to select non-zero eigenterms of the Lasso regression output for inclusion in the model and for the development of the PSA index.

To obtain the optimal disease screening model, we used five machine learning methods to build the model through the R: logistic regression (LR), random forest (RF), k-nearest neighbor (KNN), tgradient boosting (GBDT), and neural network (NN). The logistic regression and random forest models are implemented using the glmnet package and randomForest package in R, the k-nearest neighbor model is implemented via the package kknn in R, the gradient boosting model is implemented via the package xgboost in R, and the neural network model is implemented via the package neuralnet implementation. All models were built by first performing a selection of model hyperparameters, and selecting the parameter that has the best performance to build the model.

PSAII, an inflammation index developed for the first time in this study, will be developed using the results of LASSO regression and the weights of variables in the model.

#### 2.4.4 Evaluation of disease screening models, composite inflammatory index and PSAII performance

The calculation of the cutoff values was implemented through the package cutoff in R. This package calculates cutoff values that balance sensitivity and specificity. On the training set we calculated the optimal cutoff values for each model, each inflammatory index, and PSAII by using the cutoff package, and patients with screening value greater than the cutoff were considered as PSA patients.

**Figure 1:**
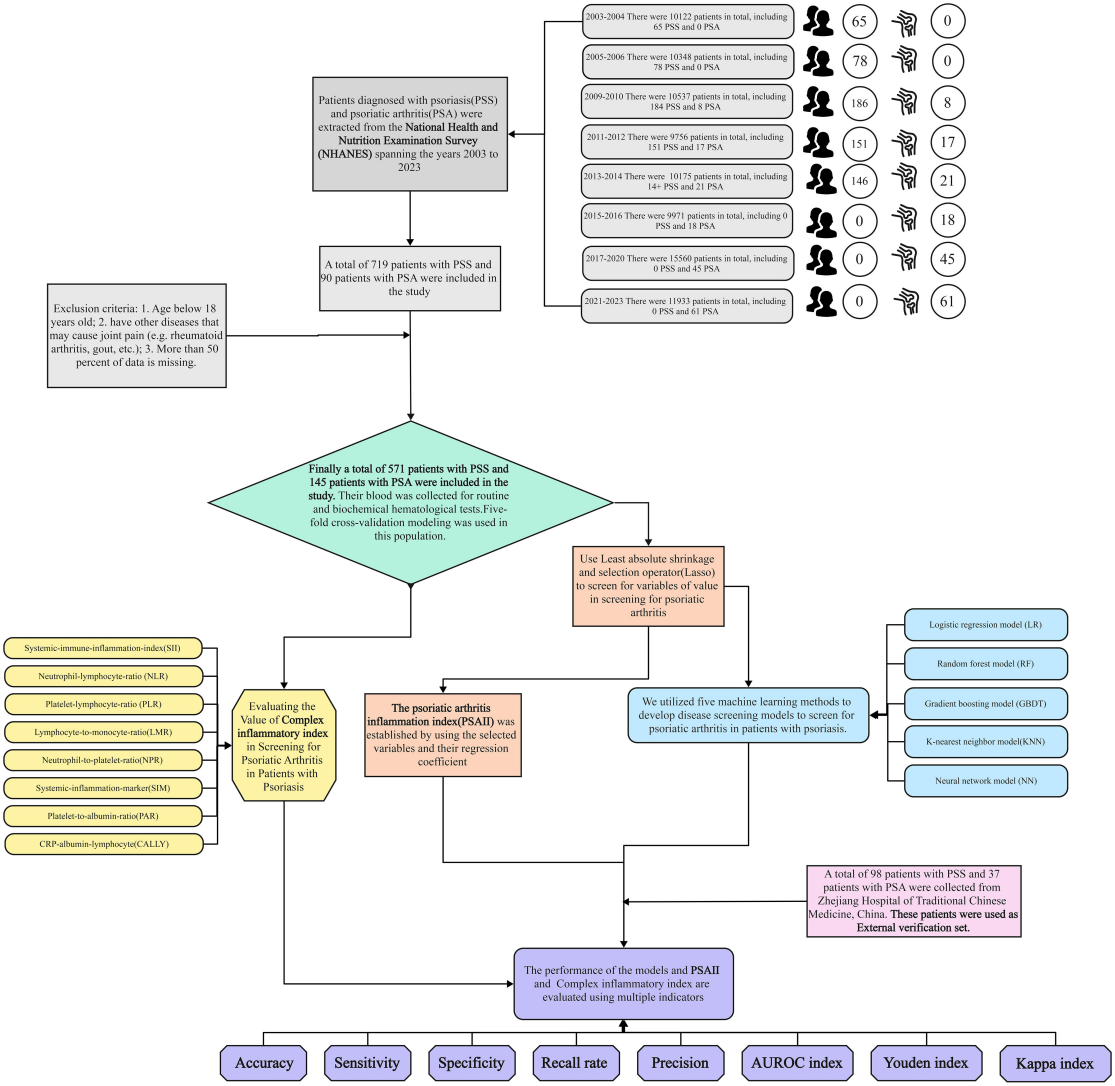
Test flow chart.

The performance evaluation of the disease screening model, composite inflammation index, and PSAII included accuracy, precision, recall, specificity, area under the curve (AUC), kappa index, Youden index, ROC curve analysis, calibration curve analysis, and decision curve analysis. Accuracy means the proportion of the total number of people been screened correctly, precision refers to the proportion of people with the disease who get a positive result, recall refers to the proportion of people with the disease who get the correct positive result, and specificity refers to the proportion of people without the disease who get a negative result. Participants above the cut-off value are considered positive results, and participants below the cut-off value are considered negative results. The closer the ROC curve is to the upper left corner, and the closer the calibration curve is to the 45-degree line between the X and Y axes, the higher the agreement between inflammation indexes and the modeled screening. To the extent that the decision curve exceeded the baseline without intervention, the net benefit of screening with models or inflammatory indicators was higher than without screening. The process of this study can be seen in **Figure 1**.

## 3 Results

### 3.1 Feature screening

**Figure 2:**
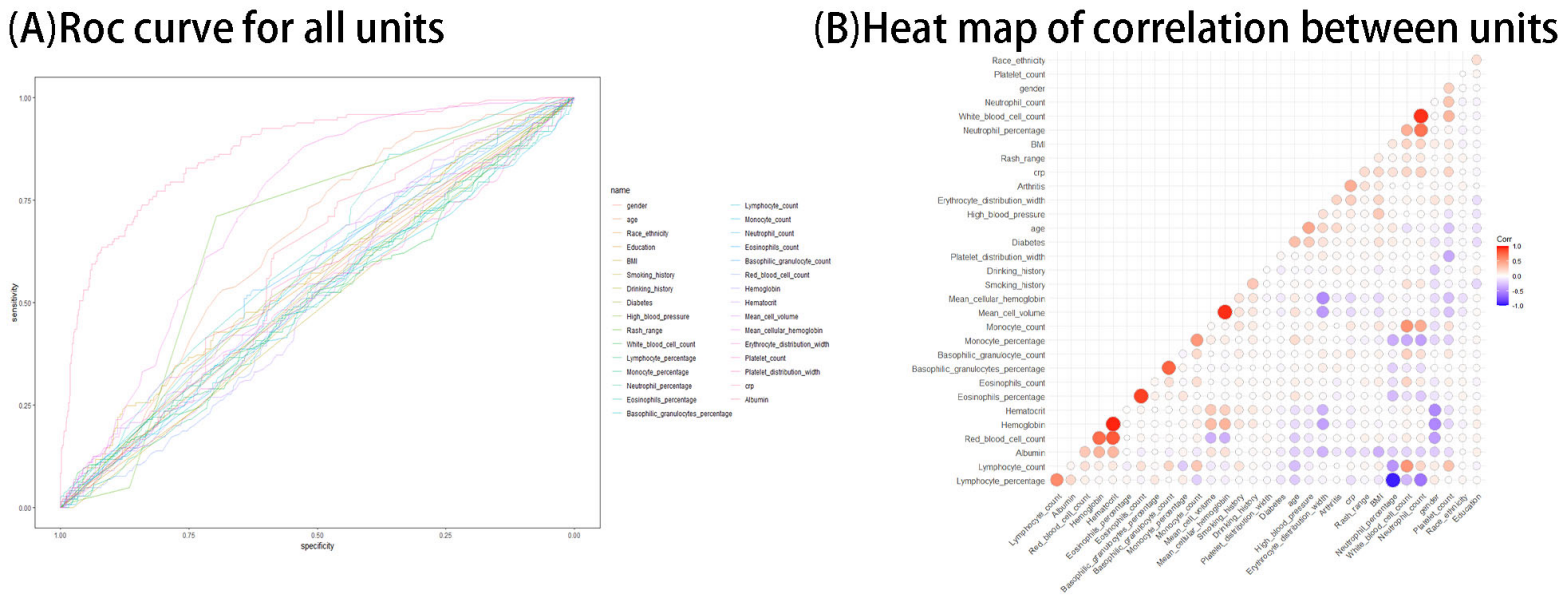
**(A, B)** ROC curve and correlation heat map of each factor.

We performed univariate analysis and ROC curve analysis and correlation analysis of all factors. The results of univariate analysis are shown in **Table 1**. The results of ROC curve and correlation test of characteristics are shown in **Figures 2A, B**. The results showed that age, mean cellular hemoglobin, erythrocyte distribution width, C-reactive protein, lymphocyte percentage, neutrophil count had the AUC values of >0.6, suggesting that they had the potential to be included in the model.

**Table 1:** Intergroup differences in psoriasis group and psoriatic arthritis group in Nhanes and Chinese population.

Variables	NHANES group (n = 719)			Chinese population group (n = 135)		
	Psoriasis group (n = 574)	Psoriatic arthritis group (n = 145)	p	Psoriasis group (n = 98)	Psoriatic arthritis group (n = 37)	p
Age (years)	48 (35,60.75)	58 (47,66)	<0.001	46.84 ± 13.89	49.24 ± 15.06	0.401
Gender, n (%)			0.385			1
Male	275 (48)	63 (43)		48 (49)	18 (49)	
Female	299 (52)	82 (57)		50 (51)	19 (51)	
Race/ethnicity, n (%)			0.074			1
Mexican American	48 (8)	10 (7)		0 (0)	0 (0)	
Other Hispanic	47 (8)	11 (8)		0 (0)	0 (0)	
Non-Hispanic White	342 (60)	75 (52)		0 (0)	0 (0)	
Non-Hispanic Black	76 (13)	33 (23)		0 (0)	0 (0)	
Other Race - Including Multi-Racial	61 (11)	16 (11)		103 (100)	32 (100)	
Education, n (%)			0.891			/
Did not graduated from junior high school	35 (6)	9 (6)		/	/	
Did not graduate high school	78 (14)	17 (12)		/	/	
High school graduate	130 (23)	33 (23)		/	/	
Some college	183 (32)	52 (36)		/	/	
College graduate	148 (26)	34 (23)		/	/	
BMI (kg/m2)	28.6 (25.26,33.49)	29.8 (25.9,34.6)	0.157	24.92 (22.28,27.68)	26.12 (23.31,28.72)	0.498
Smoking history, n (%)			0.767			/
Yes	327 (57)	80 (55)		/	/	
No	247 (43)	65 (45)		/	/	
Drinking habit, n (%)			0.055			/
Yes	447 (78)	124 (86)		/	/	
No	127 (22)	21 (14)		/	/	
Diabetes, n (%)			0.241			0.02
Yes	100 (17)	32 (22)		21 (21)	16 (43)	
No	474 (83)	113 (78)		77 (79)	21 (57)	
High blood pressure, n (%)			0.032			0.015
Yes	234 (41)	74 (51)		29 (30)	20 (54)	
No	340 (59)	71 (49)		69 (70)	17 (46)	
BSA (%)	/	/	/	7 (5,8)	8 (6,9)	0.034
Rash range, n (%)			<0.001			0.234
Little or no psoriasis	400 (70)	42 (29)		8 (8)	1 (3)	
Only a few patches	97 (17)	96 (66)		12 (12)	1 (3)	
Scattered patches	62 (11)	5 (3)		60 (61)	28 (76)	
Extensive psoriasis	15 (3)	2 (1)		18 (18)	7 (19)	
White blood cell count (10^9/L)	7.3 (5.9,8.7)	7.2 (6.1,8.76)	0.981	6.7 (5.3,8.07)	6.6 (4.8,7.2)	0.399
Lymphocyte percentage, n (%)	27.5 (23.02,33.1)	27.5 (23.1,34)	0.806	26.36 ± 7.61	32.09 ± 9.61	0.002
Monocyte percentage, n (%)	7.8 (6.5,9.1)	7.7 (6.7,9.1)	0.885	7.35 (6.23,8.8)	6.7 (5.5,7.9)	0.026
Neutrophil percentage, n (%)	61.25 (54.7,65.9)	62 (53.8,66.5)	0.814	62.68 ± 8.38	58.07 ± 10.96	0.024
Eosinophils percentage, n (%)	2.48 (1.7,3.58)	2.2 (1.4,3)	0.012	2.1 (1.2,3.2)	1.8 (1.2,3)	0.555
Basophilic granulocytes percentage, n (%)	0.6 (0.4,0.9)	0.7 (0.5,1)	<0.001	0.5 (0.4,0.7)	0.4 (0.3,0.6)	0.237
Lymphocyte count (10^9/L)	2 (1.6,2.5)	1.9 (1.6,2.4)	0.646	1.6 (1.3,2)	1.8 (1.51,1.94)	0.101
Monocyte count (10^9/L)	0.5 (0.5,0.7)	0.59 (0.4,0.7)	0.891	0.5 (0.4,0.6)	0.4 (0.35,0.42)	<0.001
Neutrophil count (10^9/L)	4.4 (3.4,5.5)	4.4 (3.3,5.58)	0.899	4.24 ± 1.4	3.48 ± 1.26	0.004
Eosinophils count (10^9/L)	0.2 (0.1,0.3)	0.2 (0.1,0.2)	0.024	0.14 (0.08,0.21)	0.11 (0.06,0.17)	0.104
Basophilic granulocyte count (10^9/L)	0 (0,0.1)	0.02 (0,0.1)	0.006	0.03 (0.02,0.05)	0.02 (0.01,0.04)	0.013
Red blood cell count (10^12/L)	4.65 ± 0.5	4.68 ± 0.47	0.553	4.55 ± 0.57	4.33 ± 0.65	0.078
Hemoglobin (g/dL)	14.2 (13.1,15.2)	14.1 (13,14.6)	0.131	14 (12.53,14.9)	12.9 (11.8,14.7)	0.056
Hematocrit, n (%)	41.57 ± 4.38	41.33 ± 3.78	0.506	40.96 (36.99,43.88)	39.78 (35.08,43.4)	0.123
Mean cell volume (fL)	89.9 (87.1,92.85)	89.6 (85.9,92.2)	0.198	90.1 (86.23,92.45)	89.8 (85.9,93.8)	0.982
Mean cellular hemoglobin (pg)	30.7 (29.33,31.7)	30.2 (28.7,31.3)	0.017	33.93 ± 0.98	33.4 ± 1.25	0.024
Erythrocyte distribution width (fL)	12.9 (12.3,13.7)	13.79 (13.3,14.4)	<0.001	13.2 (12.83,13.7)	13.3 (12.7,14)	0.686
Platelet count (10^9/L)	239 (204.25,284)	247.51 (197,285)	0.891	207 (181.25,254.75)	215 (186,300)	0.183
Platelet distribution width (fL)	8.1 (7.6,8.78)	8.3 (7.7,8.9)	0.2	8.85 (8.3,9.57)	8.8 (8.3,9.6)	0.713
C reactive protein (mg//dL)	0.43 (0.17,1.1)	4.24 (1.69,7.52)	<0.001	1.38 (1,3.77)	5.3 (1,16.55)	0.002
Albumin (g/dL)	7.3 (5.9,8.7)	7.2 (6.1,8.76)	0.981	4.02 (3.78,4.31)	4.04 (3.69,4.38)	0.917

Data are shown as mean ± standard deviation (Normal data) or Median (Q1, Q3) (Non-normal data) or n (%) (Classify data); BMI, Body Mass Index; BSA, Body surface area; NHANES, National Health And Nutrition Examination Survey.

**Figure 3:**
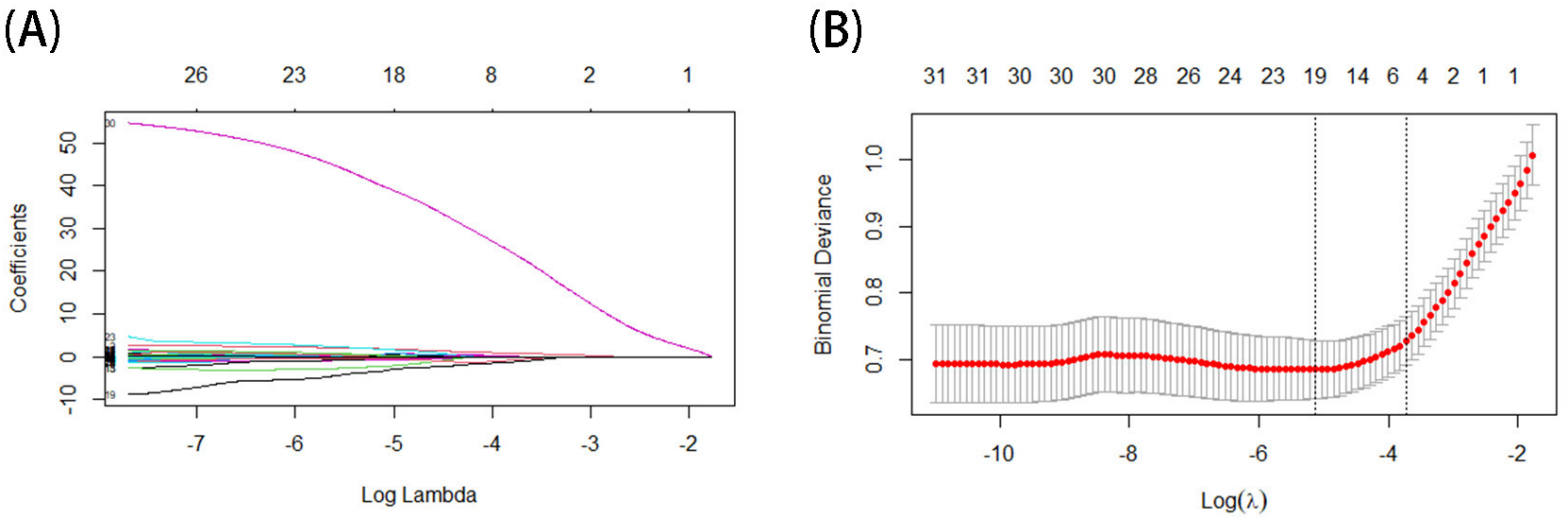
**(A, B)** The result of Lasso.

In the LASSO regression, the optimal model input parameters (lambda) were verified by 10-fold cross-validation, and their optimal values were plotted as dashed lines using the minimum standard and the standard error of the minimum standard (1-SE) (**Figures 3A, B**). In our LASSO regression results, we identified five significant variables including age, lymphocyte percentage, neutrophil count, eosinophilic count, and C-reactive protein. The correlation between these variables was minimal, so we included them all in the final model and for building the PSAII. Regression coefficients for LASSO regression can be found in **Supplementary Table 1**.

### 3.2 Comparability analysis of training and test sets

Before proceeding with the modeling, we compared the features of the NHANES database and The First Affiliated Hospital of Zhejiang Chinese Medical University database (**Table 2**). Our results show that there is some heterogeneity in the participant, which may stem from ethnic and geographical differences. This heterogeneity may be a barrier to generalizing our risk screening model and PSAII and must be carefully considered.

**Table 2:** Heterogeneity table between Nhanes group and Chinese population group.

Variables	Baseline comparison		
	Chinese population group (n = 135)	NHANES group (n = 719)	p
Age(years)	48 (35.5, 58)	51 (36, 62)	0.129
Gender, n (%)			0.434
Male	69 (51)	338 (47)	
Female	66 (49)	381 (53)	
BMI (kg/m2)	24.96 (22.57, 27.81)	28.96 (25.41, 34.22)	< 0.001
Diabetes, n (%)			0.021
Yes	37 (27)	132 (18)	
No	98 (73)	587 (82)	
High blood pressure, n (%)			0.187
Yes	49 (36)	308 (43)	
No	86 (64)	411 (57)	
Rash range, n (%)			< 0.001
Little or no psoriasis	9 (7)	442 (61)	
Only a few patches	13 (10)	193 (27)	
Scattered patches	88 (65)	67 (9)	
Extensive psoriasis	25 (19)	17 (2)	
White blood cell count(10^9/L)	6.7 (5.2, 7.9)	7.3 (5.9, 8.7)	< 0.001
Lymphocyte percentage, n (%)	26.92 (20.55, 32.75)	27.5 (23.05, 33.2)	0.493
Monocyte percentage, n (%)	7.14 (6.05, 8.5)	7.7 (6.6, 9.1)	0.005
Neutrophil percentage, n (%)	62.2 (56.2, 67.6)	61.4 (54.7, 66.1)	0.21
Eosinophils percentage, n (%)	1.9 (1.2, 3.2)	2.4 (1.6, 3.45)	0.009
Basophilic granulocytes percentage, n (%)	0.5 (0.3, 0.7)	0.6 (0.4, 0.9)	< 0.001
Lymphocyte count(10^9/L)	1.7 (1.3, 2)	2 (1.6, 2.44)	< 0.001
Monocyte count(10^9/L)	0.42 (0.39, 0.6)	0.57 (0.5, 0.7)	< 0.001
Neutrophil count(10^9/L)	3.9 (3.1, 4.85)	4.4 (3.4, 5.5)	< 0.001
Eosinophils count(10^9/L)	0.13 (0.07, 0.2)	0.2 (0.1, 0.3)	< 0.001
Basophilic granulocyte count(10^9/L)	0.03 (0.02, 0.05)	0 (0, 0.1)	< 0.001
Red blood cell count(10^12/L)	4.49 ± 0.6	4.66 ± 0.49	0.003
Hemoglobin(g/dL)	13.9 (12.4, 14.85)	14.2 (13.1, 15.09)	0.003
Hematocrit, n (%)	40.81 (36.46, 43.83)	41.78 (38.7, 44.4)	0.007
Mean cell volume(fL)	90.1 (86.1, 92.6)	89.8 (86.85, 92.71)	0.976
Mean cellular hemoglobin(pg)	33.94 (33.24, 34.56)	30.6 (29.3, 31.7)	< 0.001
Erythrocyte distribution width(fL)	13.3 (12.8, 13.8)	13.1 (12.4, 13.9)	0.09
Platelet count(10^9/L)	209 (181.5, 257.5)	240 (204, 284.5)	< 0.001
Platelet distribution width(fL)	8.8 (8.3, 9.6)	8.1 (7.6, 8.8)	< 0.001
C reactive protein(mg//dL)	1.78 (1, 6.05)	0.6 (0.21, 2.07)	< 0.001
Albumin(g/dL)	4.02 (3.77, 4.32)	4.2 (4, 4.4)	< 0.001

Data are shown as mean ± standard deviation (Normal data) or Median (Q1, Q3)(Non-normal data)or n(%)(Classify data); BMI, Body Mass Index; NHANES, National Health And Nutrition Examination Survey.

### 3.3 Performance of PSO screening models

**Figure 4:**
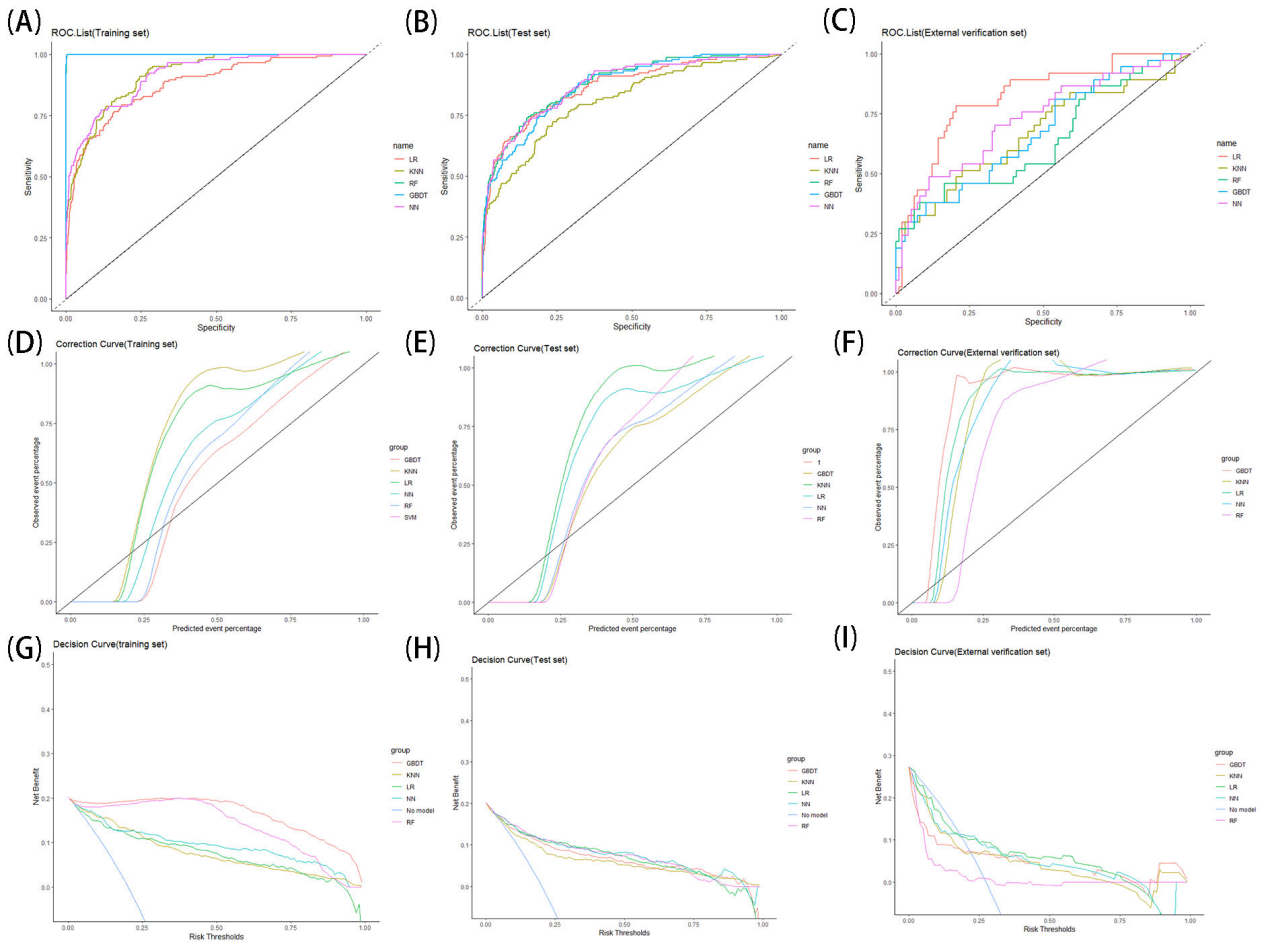
**(A–I)** ROC curve **(A–C)**, calibration curve **(D–F)** and decision curve **(G–I)** of each model on training sets, test sets and external data set.

**Figure 5:**
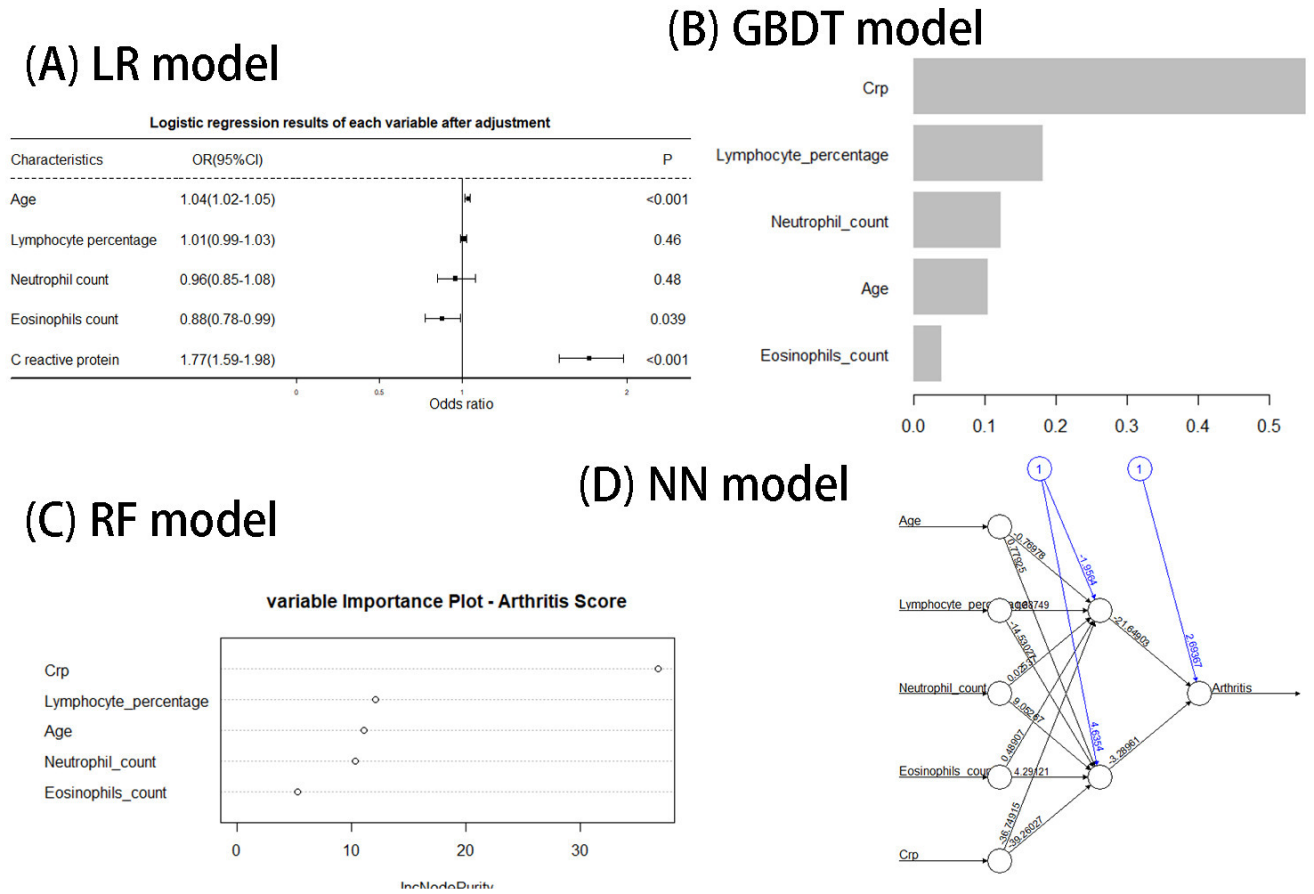
**(A–D)** The weight of each factor in the model.

In this content, we share the feature weight plots and truncation values of the models with the aim of improving the reproducibility of our models. The training, test set and external verification set’s performances of all models are shown in **Table 3**. The results of ROC curves, calibration curves, and decision curves are shown in **Figure 4**. The feature weights of each model are shown in **Figures 5A–D**, where the K-nearest-neighbor model does not have feature weights due to the specificity of its function and is therefore not shown in **Figure 5**. The confusion matrix of the model can be seen in **Supplementary Table 2**.

**Table 3:** Performance of machine learning models on training sets, test sets and external data set.

	Model	Auc	Accuracy	Precision	Recall	Specificity	Youden index	Kappa coefficient	Cutoff
Training set (n = 575)	LR	0.88(0.85-0.91)	0.81(0.78-0.84)	0.52(0.44-0.59)	0.78(0.71-0.86)	0.81(0.78-0.85)	0.33	0.50(0.43-0.58)	0.168
	KNN	0.92(0.89-0.94)	0.79(0.75-0.82)	0.49(0.42-0.55)	0.84(0.78-0.91)	0.77(0.74-0.81)	0.26	0.49(0.41-0.56)	0.157
	GBDT	0.99(0.99-1)	1(0.99-1)	0.99(0.97-1)	0.99(0.97-1)	1(0.99-1)	0.99	0.99(0.97-1)	0.388
	NN	0.92(0.89-0.94)	0.86(0.83-0.89)	0.63(0.55-0.71)	0.77(0.69-0.84)	0.88(0.86-0.91)	0.51	0.60(0.53-0.67)	0.254
	RF	0.99(0.98-1)	1(0.99-1)	0.99(0.97-1)	0.99(0.97-1)	1(0.99-1)	0.99	0.99(0.97-1)	0.387
Test set (n=144)	LR	0.87(0.83-0.90)	0.8(0.73-0.86)	0.5(0.35-0.65)	0.76(0.6-0.91)	0.81(0.74-0.88)	0.31	0.48(0.40-0.55)	//
	KNN	0.81(0.77-0.85)	0.74(0.67-0.81)	0.42(0.28-0.56)	0.72(0.56-0.89)	0.75(0.67-0.83)	0.17	0.38(0.30-0.46)	//
	GBDT	0.87(0.84-0.90)	0.85(0.79-0.91)	0.63(0.45-0.81)	0.59(0.41-0.77)	0.91(0.86-0.96)	0.54	0.51(0.42-0.59)	//
	NN	0.87(0.84-0.91)	0.83(0.77-0.89)	0.57(0.41-0.74)	0.69(0.52-0.86)	0.87(0.81-0.93)	0.44	0.52(0.44-0.60)	//
	RF	0.88(0.85-0.91)	0.86(0.8-0.92)	0.68(0.5-0.86)	0.59(0.41-0.77)	0.93(0.88-0.98)	0.61	0.56(0.48-0.64)	//
External verification set(n=135)	LR	0.82(0.74-0.90)	0.78(0.71-0.85)	0.62(0.44-0.8)	0.49(0.33-0.65)	0.89(0.83-0.95)	0.51	0.40(0.21-0.59)	
	KNN	0.67(0.56-0.78)	0.72(0.64-0.79)	0.48(0.31-0.66)	0.43(0.27-0.59)	0.83(0.75-0.9)	0.31	0.27(0.07-0.47)	
	GBDT	0.67(0.57-0.78)	0.78(0.71-0.85)	0.73(0.51-0.96)	0.3(0.15-0.44)	0.96(0.92-1)	0.69	0.31(0.10-0.53)	
	NN	0.73(0.63-0.83)	0.76(0.69-0.83)	0.6(0.41-0.79)	0.41(0.25-0.56)	0.9(0.84-0.96)	0.50	0.34(0.14-0.54)	
	RF	0.64(0.53-0.75)	0.77(0.7-0.84)	0.67(0.45-0.88)	0.32(0.17-0.48)	0.94(0.89-0.99)	0.61	0.31(0.10-0.53)	

Data are shown as n(95%CL); FN, false negatives; FP, false positives; TN, true negatives; TP, true positives; Accuracy = (TP + TN)/(TP + TN + FP + FN); Precision = TP/(TP + FP); Recall = TP/(TP + FN); Specificity = TN/(TN + FP).

#### 3.3.1 LR model

The results of the LR model on the training set show an AUC value of 0.88 (95% confidence interval (CI): 0.85-0.91), an accuracy of 0.81 (95%CI: 0.78-0.84), a precision of 0.52 (95%CI: 0.44-0.59), a recall of 0.78 (95%CI: 0.71-0.86), a specificity of 0.81 (95%CI: 0.78-0.85), and a cutoff value of 0.168; on the test set show an AUC value of 0.87 (95%CI: 0.83-0.90), an accuracy of 0.8 (95%CI: 0.73-0.86), a precision of 0.5 (95%CI: 0.35-0.65), a recall of 0.76 (95%CI: 0.6-0.91), a specificity of 0.81 (95%CI: 0.74-0.88); on the external verification set the AUC value was 0.82 (95%CI: 0.74-0.90), the precision was 0.78 (95%CI: 0.71-0.85), the accuracy was 0.62 (95%CI: 0.44-0.8), the recall was 0.49 (95%CI: 0.33-0.65), the specificity was 0.89 (95%CI: 0.83-0.95).

#### 3.3.2 KNN model

The results of the KNN model on the training set showed an AUC value of 0.92 (95%CI: 0.89-0.94), an accuracy of 0.79 (95%CI: 0.75-0.82), a precision of 0.49 (95%CI: 0.42-0.55), a recall of 0.84 (95%CI: 0.78-0.91), a specificity of 0.77 (95%CI: 0.74-0.81), and a cutoff value of 0.157; on the test set the AUC value was 0.81 (95%CI: 0.77-0.85), the accuracy was 0.74 (95%CI: 0.67-0.81), the precision was 0.42 (95%CI: 0.28-0.56), the recall was 0.72 (95%CI: 0.56-0.89), the specificity was 0.75 (95%CI: 0.67-0.83); on the external verification set the AUC value was 0.67 (95%CI: 0.56-0.78), the accuracy was 0.72 (95%CI: 0.64-0.79), the precision was 0.48 (95%CI: 0.31-0.66), the recall was 0.43 (95%CI: 0.27-0.59), the specificity was 0.83 (95%CI: 0.75-0.9).

#### 3.3.3 GBDT model

The results of the GBDT model on the training set showed an AUC value of 0.99 (95%CI: 0.99-1), an accuracy of 1 (95%CI: 0.99-1), a precision of 0.99 (95%CI: 0.97-1), a recall of 0.99 (95%CI: 0.97-1), a specificity of 1 (95%CI: 0.99-1), and a cutoff value of 0.388; on the test set the AUC value was 0.87 (95%CI: 0.84-0.90), the accuracy was 0.85 (95%CI: 0.79-0.91), the precision was 0.63 (95%CI: 0.45-0.81), the recall was 0.59 (95%CI: 0.41-0.77), the specificity was 0.91 (95%CI: 0.86-0.96); on the external verification set the AUC value was 0.67 (95%CI: 0.57-0.78), the accuracy was 0.78 (95%CI: 0.71-0.85), the precision was 0.73 (95%CI: 0.51-0.96), the recall was 0.3 (95%CI: 0.15-0.44), the specificity was 0.96 (95%CI: 0.92-1).

#### 3.3.4 NN model

The results of the NN model on the training set showed an AUC value of 0.92 (95%CI: 0.89-0.94), an accuracy of 0.86 (95%CI: 0.83-0.89), a precision of 0.63 (95%CI: 0.55-0.71), a recall of 0.77 (95%CI: 0.69-0.84), a specificity of 0.88 (95%CI: 0.86-0.91), and a cutoff value of 0.254; on the test set the AUC value was 0.87 (95%CI: 0.84-0.91), the accuracy was 0.83 (95%CI: 0.77-0.89), the precision was 0.57 (95%CI: 0.41-0.74), the recall was 0.69 (95%CI: 0.52-0.86), the specificity was 0.87 (95%CI: 0.81-0.93); on the external verification set the AUC value was 0.73 (95%CI: 0.63-0.83), the accuracy was 0.76 (95%CI: 0.69-0.83), the precision was 0.6 (95%CI: 0.41-0.79), the recall was 0.41 (95%CI: 0.25-0.56), the specificity was 0.9 (95%CI: 0.84-0.96).

#### 3.3.5 RF model

The results of the RF model on the training set showed an AUC value of 0.99 (95%CI: 0.99-1), an accuracy of 1 (95%CI: 0.99-1), a precision of 0.99 (95%CI: 0.97-1), a recall of 0.99 (95%CI: 0.97-1), a specificity of 1 (95%CI: 0.99-1), and a cutoff value of 0.387; on the test set the AUC value was 0.88 (95%CI: 0.85-0.91), the accuracy was 0.86 (95%CI: 0.8-0.92), the precision was 0.68 (95%CI: 0.5-0.86), the recall was 0.59 (95%CI: 0.41-0.77), the specificity was 0.93 (95%CI: 0.88-0.98); on the external verification set the AUC value was 0.64 (95%CI: 0.53-0.75), the accuracy was 0.77 (95%CI: 0.7-0.84), the precision was 0.67 (95%CI: 0.45-0.88), the recall was 0.32 (95%CI: 0.17-0.48), the specificity was 0.94 (95%CI: 0.89-0.99).

### 3.4 Evaluating the screening potential of the composite inflammatory index and PSAII

In this element, we performed a univariate analysis of the composite inflammatory indices (see **Table 4**), PSAII and CALLY had a p<0.05 on both the NHANES group and Chinese population group, and were considered by us to have potential for screening PSA.

**Table 4:** Characteristic table of existing inflammatory indicators and Psoriatic arthritis inflammation index in psoriatic arthritis and psoriasis.

Variables	Nhanes group (n = 719)			Chinese population group (n = 135)		
	Psoriasis group (n = 574)	Psoriatic arthritis group (n = 145)	p	Psoriasis group (n = 98)	Psoriatic arthritis group (n = 37)	p
Systemic immune inflammation index (SII)	530.29 (373.5, 736.55)	558.09 (363.6, 738)	0.91	514.15 (364.32, 655.62)	377.01 (249.47, 741.11)	0.211
Neutrophil lymphocyte ratio (NLR)	2.22 (1.66, 2.9)	2.3 (1.63, 2.9)	0.926	2.49 (1.89, 3.34)	1.91 (1.37, 2.44)	0.005
Platelet lymphocyte ratio (PLR)	122.72 (97.18, 153.12)	124.62 (101.11, 155)	0.776	128.65 (102.62, 158.57)	123.33 (99.21, 171.25)	0.721
Lymphocyte to monocyte ratio (LMR)	3.6 (2.8, 4.6)	3.62 (2.73, 4.67)	0.915	3.33 (2.67, 4.33)	4.75 (3.7, 5.6)	< 0.001
Neutrophil to platelet ratio (NPR)	0.02 (0.01, 0.02)	0.02 (0.01, 0.02)	0.926	0.02 (0.01, 0.02)	0.01 (0.01, 0.02)	< 0.001
Systemic inflammation marker (SIM)	1.21 (0.82, 1.71)	1.26 (0.78, 1.75)	0.926	1.19 (0.78, 1.7)	0.68 (0.5, 1.13)	< 0.001
Platelet to albumin ratio (PAR)	56.74 (47.45, 67.53)	60.83 (46.83, 69.53)	0.275	53.15 (42.99, 62.55)	49.46 (43.86, 75.29)	0.445
CRP albumin lymphocyte (CALLY)	1.93 (0.75, 5.03)	0.22 (0.1, 0.47)	< 0.001	0.43 (0.16, 0.79)	0.16 (0.03, 0.65)	0.011
Psoriatic arthritis inflammation index (PSAII)	1.44 (0.59, 3.93)	16.88 (5.95, 33.75)	< 0.001	8.95 (3.83, 18.70)	30.00 (19.72, 146.31)	< 0.001

Data are shown as mean ± standard deviation (Normal data) or Median (Q1, Q3)(Non-normal data)or n(%)(Classify data); SII, Platelet count × neutrophil count/lymphocyte count; NLR, Neutrophil count/lymphocyte count; PLR, Platelet count/lymphocyte count; LMR, Lymphocyte count/monocyte count; NPR, Neutrophil count/platelet count; SIM, Monocyte count × neutrophil count/lymphocyte count; PAR, Platelet count/albumin; CALLY, Albumin × Lymphocyte/(C-reactive protein ×10); PSAII, (Lymphocyte percentage × C-reactive protein)/(Neutrophil count × Eosinophilic counts ×10).

The PSAII was developed based on the results of the LASSO regression and the weights of LR model (see **Figure 5**), with metrics with risk ratios less than 1 placed under the divisor and metrics with risk ratios greater than 1 placed over the divisor. The final PSAII was defined as PSAII = percentage of lymphocytes × C-reactive protein (mg/dL)/(neutrophil count (10^9/L) ×eosinophilic counts (10^9/L) ×10).

**Figure 6:**
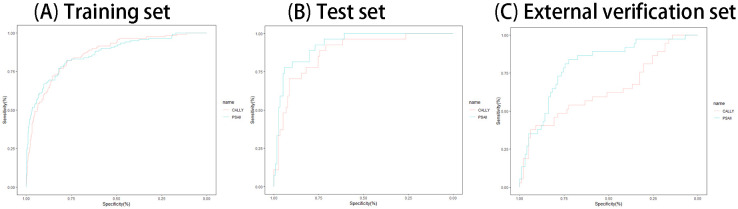
**(A–C)** The ROC curves of all composite inflammation metrics for both training sets **(A)**, test sets **(B)** and external data set **(C)**.

Participants with PSAII metrics greater than the cutoff value were considered to have PSA, and those with CALLY metrics less than the cutoff value were considered to have PSA. The performance of all composite inflammation metrics is shown in **Table 5**. The ROC curves of all composite inflammation metrics for both the training and the test sets and external verification set are shown in **Figures 6A–C**.

**Table 5:** Performance of inflammation index in training sets, test sets and external data set.

	Index	Auc	Accuracy	Precision	Recall	Specificity	Youden index	Kappa coefficient	Cutoff
Training set (n = 585)	CRP albumin lymphocyte (CALLY)	0.86(0.82-0.90)	0.78(0.75-0.82)	0.48(0.41-0.55)	0.82(0.75-0.89)	0.77(0.73-0.81)	0.26	0.47(0.39-0.55)	0.61
	Psoriatic arthritis inflammation index (PSAII)	0.86(0.83-0.90)	0.87(0.84-0.9)	0.81(0.72-0.9)	0.47(0.38-0.56)	0.97(0.96-0.99)	0.78	0.52(0.42-0.62)	18
Test set (n=144)	CRP albumin lymphocyte (CALLY)	0.87(0.81-0.94)	0.8(0.73-0.86)	0.48(0.33-0.62)	0.78(0.62-0.93)	0.8(0.73-0.88)	0.28	0.47(0.29-0.64)	//
	Psoriatic arthritis inflammation index (PSAII)	0.93(0.88-0.97)	0.88(0.82-0.93)	0.8(0.6-1)	0.44(0.26-0.63)	0.97(0.95-1)	0.77	0.51(0.29-0.72)	//
External verification set (n=135)	CRP albumin lymphocyte (CALLY)	0.64(0.53-0.75)	0.43(0.35-0.51)	0.28(0.19-0.37)	0.68(0.52-0.83)	0.34(0.24-0.43)	-0.39	0.08(-0.14-0.15)	
	Psoriatic arthritis inflammation index (PSAII)	0.81(0.72-0.89)	0.76(0.68-0.83)	0.54(0.41-0.67)	0.81(0.68-0.94)	0.73(0.65-0.82)	0.27	0.47(0.31-0.63)	

Data are shown as n(95%CL); FN, false negatives; FP, false positives; TN, true negatives; TP, true positives; Accuracy = (TP + TN)/(TP + TN + FP + FN); Precision = TP/(TP + FP); Recall = TP/(TP + FN); Specificity = TN/(TN + FP).

#### 3.4.1 CALLY

The results of CALLY on the training set showed an AUC value of 0.86 (95%CI: 0.82-0.90), an accuracy of 0.78 (95%CI: 0.75-0.82), a precision of 0.48 (95%CI: 0.41-0.55), a recall of 0.82 (95%CI: 0.75-0.89), a specificity of 0.77 (95%CI: 0.73-0.81), and a cutoff value of 0.61; on the test set the AUC value was 0.87 (95%CI: 0.81-0.94), the accuracy was 0.8 (95%CI: 0.73-0.86), the precision was 0.48 (95%CI: 0.33-0.62), the recall was 0.78 (95%CI: 0.62-0.93), the specificity was 0.8 (95%CI: 0.73-0.88); on the external verification set the AUC value was 0.64 (95%CI: 0.53-0.75), the accuracy was 0.43 (95%CI: 0.35-0.51), the precision was 0.28 (95%CI: 0.19-0.37), the recall was 0.68 (95%CI: 0.52-0.83), the specificity was 0.34 (95%CI: 0.24-0.43).

#### 3.4.2 PSAII

The PSAII evaluation results on the training set showed an AUC value of 0.86 (95%CI: 0.83-0.90), an accuracy of 0.87 (95%CI: 0.84-0.9), a precision of 0.81 (95%CI: 0.72-0.9), a recall of 0.47 (95%CI: 0.38-0.56), a specificity of 0.97 (95%CI: 0.96-0.99), and a cutoff value of 18; on the test set the AUC value was 0.93 (95%CI: 0.88-0.97), the accuracy was 0.88 (95%CI: 0.82-0.93), the precision was 0.8 (95%CI: 0.6-1), the recall was 0.44 (95%CI: 0.26-0.63), the specificity was 0.97 (95%CI: 0.95-1); on the external verification set the AUC value was 0.81 (95%CI: 0.72-0.89), the accuracy was 0.76 (95%CI: 0.68-0.83), the precision was 0.54 (95%CI: 0.41-0.67), the recall was 0.81 (95%CI: 0.68-0.94), the specificity was 0.73 (95%CI: 0.65-0.82).

**Figure 7:**
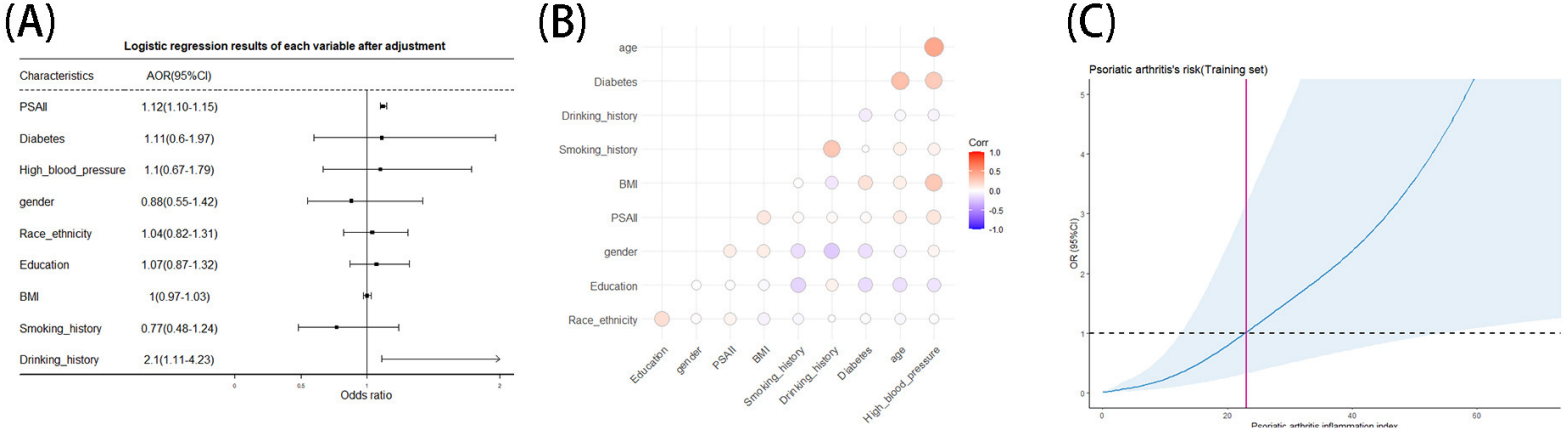
**(A–C)** PSAII's logistic regression **(A)**, correlation heat maps **(B)** and restricted cubic spline analysis **(C)**.

Besides, we analyzed the relationship between PSAII and covariates. Logistic regression results (**Figure 7A**) showed that PSAII (p<0.001, OR=1.12, 95%CI:1.10-1.15) was an independent risk factor for PSA after adjusting for age, gender, BMI, drinking history, smoking history, education, diabetes history, and hypertension history. Correlation analysis (**Figure 7B**) showed that PSAII had little correlation with other covariables. In addition, we performed a restricted cubic spline analysis of PSAII (**Figure 7C**), which showed that after adjusting for age, gender, race, drinking history, smoking history, education, diabetes history, hypertension history and BMI, PSAII and PSA were nonlinearly related. Meanwhile the risk of PSA progressively increased as the PSAII index increased.

## 4 Discussion

When comparing the various models, both GBDT and RF models perform very well on the training set, outperforming the other models in terms of ROC value, accuracy, precision, recall and specificity, however, on the test set the two models do not perform as well as they should, which may stem from the overfitting of the GBDT and RF models on the training set. On the test set and external verification set, consider both the Kappa value and the Youden index together that the LR and NN models perform well with good ROC values, accuracy, precision, recall, and specificity, and maintain good performance in ROC curve, calibration curve, and decision curve analysis.

Combining the performance of the test sets and external verification set, we believe that the LR model is the best model for screening PSA in our study. As the most widely used model, the LR model has excellent classification performance. The logistic regression model takes the natural logarithm of the odds as a regression function of the predictors. With 1 predictor, X, this takes the form ln[odds(Y=1)] = β0+β1X, where ln stands for the natural logarithm, Y is the outcome and Y=1 when the event happens (29). The LR model we have developed can screen PSA well, thus enabling PSA patients to be diagnosed and treated correctly and in a timely manner.

Even though screening models built by machine learning may have better screening efficiency, these models cannot be conveniently used in the clinic. This is why we are committed to developing PSAII. Compared to other composite inflammation indices, both CALLY and PSAII performed better on the training set. However, on the external verification set, CAALY’s performance declined a lot, but PSAII maintained its better performance. Meanwhile, the restrictive cubic spline analysis of PSAII showed a gradual increase in the risk of PSA as the PSAII index rose, which indicates that PSAII has good screening potential.

The PSAII we developed still performs well in the face of external data, significantly outperforming other composite inflammation indices, and it is more economical and convenient as it requires only routine blood data, so we believe it has the potential for wide-scale dissemination. It is worth noting that the PSAII we developed performs better in terms of accuracy, recall and specificity, which implies that it excels in disease screening. However, it performs poorly in terms of precision, which means that PSAII has a high false-positive rate and is limited in its ability to accurately diagnose PSA, which needs to be accompanied by other diagnostic tools to confirm the PSA diagnosis.

**Figure 8:**
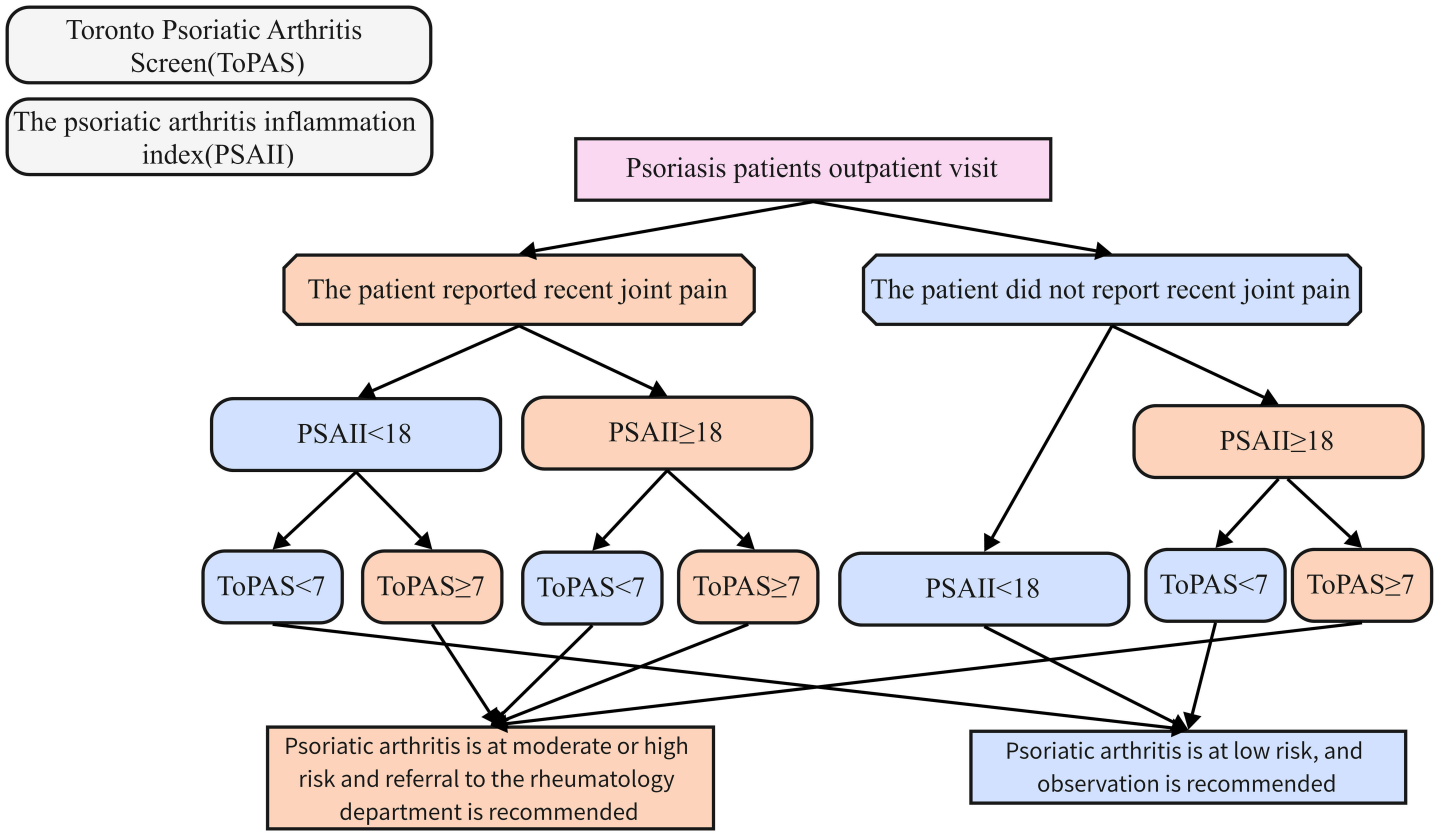
Combined PSA Screening strategies from PSAII and the Toronto Psoriasis Arthritis Screening (ToPAS).

Accordingly, we try to propose a PSA screening strategy that combines PSAII and the Toronto Psoriasis Arthritis Screening (ToPAS) (**Figure 8**): PSO patients are first divided into arthralgia and non-arthralgia groups according to the presence or absence of complaints of joint pain at the time of consultation. Patients in the non-arthralgia group who have a PSAII of less than 18 or a ToPAS of less than 7 are admitted to the low-risk group for PSA, the rest of the patients in the non-arthralgia group were included in the high-risk group for PSA. Patients in the arthralgia group who have a PSAII greater than or equal to 18 or a ToPAS greater than or equal to 7 are entered into the PSA high-risk group, the rest of the patients arthralgia group were included in the low-risk group for PSA. Patients in the high-risk group for PSA are advised to take more proactive diagnostic and treatment measures. In our proposed PSA screening system, we emphasize the judgment of PSAII index first. This is because PSAII is an objective index, subject to less subjective interference, obtained in a convenient manner without delaying outpatient consultation time, which can improve the efficiency of PSA screening and reduce the rate of PSA leakage. In future studies, we plan to conduct a multicenter cohort study to validate the feasibility of our proposed PSA screening strategy.

In our study, five metrics were included in the screening model, including age (OR: 1.04), lymphocyte ratio (OR: 1.01), neutrophil count (OR: 0.96), C-reactive protein (OR: 1.77), and eosinophilic count (OR: 0.88) (the logistic regression results can be seen in **Figure 5A**). We will discuss the effects of each of these factors on PSA in turn.

In our study it was suggested that advanced age is an independent risk factor for PSA, many studies have come to similar conclusions as ours (30, 31). This may be due to the accumulation of PSO and its various comorbidities with age, which activate inflammatory or non-inflammatory pathways leading to the development of PSA (32). In addition, obesity and psychological disorders due to aging may also have an impact on the development of PSA (33).

C-reactive protein is a non-specific indicator of inflammation. As seen in **Figure 5**, the contribution of C-reactive protein is the largest in all model. The relationship between systemic inflammation and CRP is complex, with C-reactive protein levels correlating with the risk of cardiovascular disease, diabetes, metabolic syndrome, rheumatoid arthritis, lung disease, and depression (34). CRP is synthesized mainly in liver cells, there is growing evidence that CRP plays an important role in inflammatory processes and host response to infection, including the complement pathway, apoptosis, and the production of cytokines, particularly interleukin-6 (IL-6) and tumor necrosis factor-α (35). Tumor necrosis factor-α is a cytokine highly expressed in psoriatic lesions, which induces inflammation by acting on keratinocytes mainly through two types of tumor necrosis factor receptors (36). Studies have shown that IL-6 plays an active role in initiating T helper 17 cell development, which may aggravate psoriasis inflammation (37). In clinical Settings, many studies have pointed to a strong correlation between C-reactive protein levels and the severity of PSO and PSA (38, 39). Therefore, many studies have also advocated the use of C-reactive protein as an indicator to monitor the activity of PSA (40, 41).

The elevated percentage of lymphocytes may reflect activation of inflammatory pathways, with T lymphocytes receiving interleukin(IL)-23 released by dendritic cells and releasing IL-17 (42), IL-17 binds to receptors on keratinocytes and stimulates keratinocytes to release inflammatory factors such as TNF-α (43), this is a central pathway in the pathogenesis of PSO and PSA (14). In addition to this, in the emerging field of the innate immune system, innate lymphoid cells (ILC) 3 in ILC appear to be important in psoriasis because of their ability to produce IL-22 and IL-17A. Patients have higher levels of ILC3 in their blood and skin compared to healthy people. In addition, their numbers in peripheral blood and affected skin decreased with disease remission, suggesting that cell numbers were inversely correlated with disease activity (44). In clinical practice, although no direct use of lymphocyte ratio screening and detection of PSA has been reported. A number of complex inflammatory indicators including lymphocytes, including NLR, PLR, MLR, are widely used to detect PSO severity (45). This reflects the close relationship between lymphocytes and PSA.

Typically, activation of neutrophils in serum and skin tissues is a distinguishing feature of PSO (46). Neutrophils are closely associated with psoriasis. The production of reactive oxygen species, degranulation and formation of neutrophil extracellular traps are the main attack functions of neutrophils, which contribute to the immune pathogenesis of PSO and PSA (47–49). More importantly, neutrophils are one of the main cellular sources of IL-17 production in PSO and PSA (50). Our study showed that PSA patients had fewer neutrophils in their serum than PSO patients. A possible reason for this is the increased recruitment of neutrophils into joint tissues which leads to a decrease in serum neutrophils (51). One study proposed that reducing neutrophil recruitment could be effective in reducing symptoms of PSO and PSA (52). However, the detailed molecular mechanisms of neutrophil chemotaxis and activation remain unclear, and our conclusions need to be validated by rigorous experiments.

In our study, patients with PSA had lower eosinophil counts than those with PSO, which had not been mentioned in previous studies. Eosinophils are commonly thought to be involved in a variety of infectious, allergic, and autoimmune diseases through degranulation (53). However, with the deepening of the study of eosinophils, more and more functions have been discovered. Several studies have shown that different subgroups of eosinophils have pro-inflammatory and pro-inflammatory regression properties, respectively (54). In an animal study, it was demonstrated that eosinophils are recruited to the site of inflammation as coordinators of inflammation resolution and induce differentiation of macrophages from a pro-inflammatory M1 phenotype to an anti-inflammatory M2 phenotype by secreting IL-4, IL-13, and 12/15-Lox-derived lipid mediators (55). From this, we suppose that in PSO, less circulating blood levels of eosinophils led to more severe inflammation, which affects the onset of PSA. One study on rheumatoid arthritis (RA) was similar to our view, and their findings noted that circulating eosinophile counts were inversely associated with disease activity index in RA patients and increased after receiving anti-rheumatic therapy (56). However, the role of eosinophils in psoriatic arthritis remains unclear, and our hypothesis needs to be validated by cell and animal studies and large cohort studies.

There are some limitations to this study. First, because of the limitations of the NHANES database, we were unable to know the disease severity of PSA patients and whether PSA would occur in the future in PSO patients, which limits the potential of our model and PSAII in the direction of predicting PSA onset and monitoring PSA disease activity. At the same time, limited by the NHANES database, we can only diagnose PSO and PSA based on patients’ self-reports, which may have recall bias. Such diagnostic criteria lack specificity. Second, because of the lack of data on drug therapy, this study did not consider the effect of therapeutic drugs received by participants from the NHANES database on serum markers in patients with PSO and PSA, which may affect the reliability of the conclusions. Finally, our study only included population-based data from the United States and China, and caution should be exercised when expanding to external datasets, and some of our conclusions need to be validated by additional high-quality studies.

Even so, this study has some strengths. First, the PSAII we developed continues to perform well in external validation sets across ethnic and geographic regions, indicating its potential for generalization. Second, we excluded the confounding factor of medication from the patients collected at The First Affiliated Hospital of Zhejiang University of Traditional Chinese Medicine as a complement to NHANES to try our best to minimize the limitations described in the previous section.

## 5 Conclusion

PSA is with an insidious onset and a high rate of missed diagnosis, and delayed diagnosis can lead to its poor prognosis. Therefore, it is of great interest to screen PSA in PSO patients. We developed and validated five disease screening models using machine learning algorithms to screen PSO patients for PSA by analyzing serum data from NHANES and Chinese populations, and found that the logistic regression algorithm had the best performance after comparison. In addition, this study developed PSAII that can be used in an outpatient setting to aid in PSA screening based on the results of model and LASSO regression, which performed well in both the US and Chinese populations. However, the high false positive rate of PSAII makes it necessary to combine it with other PSA screening tools when applied.

## Data Availability

The original contributions presented in the study are included in the article/**Supplementary Material**. Further inquiries can be directed to the corresponding authors.
