# Supplementary material for: Psoriatic arthritis in psoriasis: optimizing the current screening system for psoriatic arthritis based on serum data from U.S. and Chinese populations
Source: Front Immunol. 2024 Dec 10;15:1497713. doi: 10.3389/fimmu.2024.1497713 (PMC11666430; doi:10.3389/fimmu.2024.1497713)
Supplement: Supplementary file 1 [file Table1.docx]

Supplement 1：Results of LASSO regression

| Variable | Coefficient of lasso regression |
| --- | --- |
| (Intercept) | -2.20192 |
| Gender | / |
| Age | 0.827656 |
| Race ethnicity | / |
| Education | / |
| BMI | / |
| Smoking history | / |
| Drinking history | / |
| Diabetes | / |
| High blood pressure | / |
| Rash range | / |
| White blood cell count | / |
| Lymphocyte percentage | 0.080738 |
| Monocyte percentage | / |
| Neutrophil percentage | / |
| Eosinophils percentage | / |
| Basophilic granulocytes percentage | / |
| Lymphocyte count | / |
| Monocyte count | / |
| Neutrophil count | -1.07508 |
| Eosinophils count | -0.63895 |
| Basophilic granulocyte count | / |
| Red blood cell count | / |
| Hemoglobin | / |
| Hematocrit | / |
| Mean cell volume | / |
| Mean cellular hemoglobin | / |
| Erythrocyte distribution width | / |
| Platelet count | / |
| Platelet distribution width | / |
| C-reactive protein | 23.39691 |
