# Supplementary material for: Psoriatic arthritis in psoriasis: optimizing the current screening system for psoriatic arthritis based on serum data from U.S. and Chinese populations
Source: Front Immunol. 2024 Dec 10;15:1497713. doi: 10.3389/fimmu.2024.1497713 (PMC11666430; doi:10.3389/fimmu.2024.1497713)
Supplement: Supplementary file 2 [file Table2.docx]

Supplementary material 2 Confusion matrix of five models and inflammation index

|  | Model or index | TN | FN | FP | TP |
| --- | --- | --- | --- | --- | --- |
| Training set (n = 585) | LR | 374 | 25 | 85 | 91 |
|  | KNN | 355 | 18 | 104 | 98 |
|  | GBDT | 458 | 1 | 1 | 115 |
|  | NN | 406 | 27 | 53 | 89 |
|  | RF | 458 | 1 | 1 | 115 |
|  | CRP albumin lymphocyte (CALLY) | 353 | 21 | 104 | 97 |
|  | Psoriatic arthritis inflammation index (PSAII) | 444 | 63 | 13 | 55 |
| Test set (n=144) | LR | 93 | 7 | 22 | 22 |
|  | KNN | 86 | 8 | 29 | 21 |
|  | GBDT | 105 | 12 | 10 | 17 |
|  | NN | 100 | 9 | 15 | 20 |
|  | RF | 107 | 12 | 8 | 17 |
|  | CRP albumin lymphocyte (CALLY) | 94 | 6 | 23 | 21 |
|  | Psoriatic arthritis inflammation index (PSAII) | 114 | 15 | 3 | 12 |
| External verification set (n=135) | LR | 87 | 19 | 11 | 18 |
|  | KNN | 81 | 21 | 17 | 16 |
|  | GBDT | 94 | 26 | 4 | 11 |
|  | NN | 88 | 22 | 10 | 15 |
|  | RF | 92 | 25 | 6 | 12 |
|  | CRP albumin lymphocyte (CALLY) | 33 | 12 | 65 | 25 |
|  | Psoriatic arthritis inflammation index (PSAII) | 72 | 7 | 26 | 30 |

FN, false negatives; FP, false positives; TN, true negatives; TP, true positives.
